# Automating weighing of faces and voices based on cue saliency in trustworthiness impressions

**DOI:** 10.1038/s41598-023-45471-y

**Published:** 2023-11-16

**Authors:** Marc-Lluís Vives, Candice Frances, Cristina Baus

**Affiliations:** 1 Institute of Psychology, Leiden University, Leiden, The Netherlands; 2 Psychology of Language, Max Planck Institute for Psycholinguistics, Nijmegen, The Netherlands; 3 Department of Cognition, Development and Educational Psychology, University of Barcelona, Barcelona, Spain

**Keywords:** Psychology, Human behaviour

## Abstract

When encountering people, their faces are usually paired with their voices. We know that if the face looks familiar, and the voice is high-pitched, the first impression will be positive and trustworthy. But, how do we integrate these two multisensory physical attributes? Here, we explore 1) the automaticity of audiovisual integration in shaping first impressions of trustworthiness, and 2) the relative contribution of each modality in the final judgment. We find that, even though participants can focus their attention on one modality to judge trustworthiness, they fail to completely filter out the other modality for both faces (Experiment 1a) and voices (Experiment 1b). When asked to judge the person as a whole, people rely more on voices (Experiment 2) or faces (Experiment 3). We link this change to the distinctiveness of each cue in the stimulus set rather than a general property of the modality. Overall, we find that people weigh faces and voices automatically based on cue saliency when forming trustworthiness impressions.

## Introduction

People are multidimensional and multimodal. We can judge others based on a vast array of physical attributes, such as how they look or the tone of their voice. When meeting new people, our visual system provides us with detailed information about people’s looks, which is rapidly transformed into a trustworthiness signal^1^. If the face looks familiar^2^ or has a certain physiognomy^3^, the trustworthiness signal is positive. But sometimes, a trustworthy face is accompanied by a negative signal originating from another sense. Every now and then, we meet people who elicit ambiguity: they look trustworthy, but they sound untrustworthy, or vice versa. How do we solve the ambiguity caused by this multisensory mismatch? That is, how are multisensory cues integrated to inform first impressions?

Most research has focused on understanding first impressions originated by physical cues from one perceptual system, mainly the visual one. Through the visual stream, people judge facial trustworthiness very rapidly (in less than 200 milliseconds (ms)^4,5^), automatically, and with high consistency across people. Through audition, people also reliably judge trustworthiness based solely on the voice of the person^6^. With a simple and short “Hello!” people can assess and rate the trustworthiness of a person^7^, even when listening to foreign-accented voices^8^. Here, we assess how people integrate visual and auditory cues to judge the trustworthiness of a person.

Multisensory integration automatically evokes a set of unique questions. First, which channel drives people’s first impressions the most, the visual or the auditory stream? Are people’s trustworthiness judgments mostly driven by the face or the voice of a person? Previous research has shown that, for trustworthiness, faces are more important than voices^9,10^. However, the reason for this face-primacy effect in the audiovisual integration of trustworthiness information is unknown. Here, we seek a parsimonious explanation based on the perceptual properties of the stimulus. We hypothesize that the cue that is weighted more heavily depends on stimulus saliency, which is determined by the overall distinctiveness of a stimulus in the stimuli set. That is, faces presented in a homogenous set will be down-weighted in trustworthiness judgments compared to voices within a group with higher vocal variability, and vice versa. In more general terms, the weight of the cue depends on its discriminability.

A second open question refers to whether audiovisual integration is automatic^5^. People’s judgments are, in fact, affected by information from both channels even when they are asked to ignore one of them^9^. But, it is still unknown whether that still occurs when faces and voices are presented for a very brief amount of time—e.g., 500 ms. Audiovisual integration is very fast, already occurring in 100 ms, which might make it hard to filter one modality. Here, we investigate whether people can overcome the influence of one of the channels with temporally very limited exposure. In turn, this provides an indirect estimate of the time needed to join audiovisual cues in personality trait judgments.

We address these questions by running four experiments. First, we manipulate whether participants need to judge only the voice (Experiment 1a), the face (Experiment 1b), or both (Experiment 2). We then change the face stimuli to test for the saliency hypothesis (Experiment 3). Overall, we find that faces’ and voices’ trustworthiness are automatically weighted based on saliency to inform first impressions.

## General methods

### Participants

In Experiments 1–3, 181 participants were recruited using Prolific. Based on previous work, we expected a relatively large effect size (omega-squared = 0.09). We ran an a priori power analysis and determined that a sample of 25 participants was needed to obtain 90% power. Experiments 2 and 3 were pre-registered (https://aspredicted.org/GYT_AZP; https://aspredicted.org/FMD_95Y). The following exclusion criteria were established prior to the experiment (see pre-registrations): giving the same answer on at least 80% of the trials or failing the attentional check. The attentional check consisted of reporting what participants saw and heard in the last trial of the experiment, included only for this purpose. We ran enough participants to compensate for a 10–20% dropout rate to meet these criteria in each experiment. Before participating all participants gave informed consent, and they were paid after completing the study. All methods were in accordance with the guidelines of the ethics committee at Pompeu Fabra University and approval of the experimental protocol was obtained from the university’s ethics committee (CEIC-*Comité Étic d’Investigació Clínica*).

### Experimental design

Faces and voices were paired in a full two-by-two within-subjects design: congruent trustworthy (trustworthy face and voice), congruent untrustworthy (untrustworthy face and voice), and incongruent (trustworthy face with an untrustworthy voice and vice versa). Participants completed a total of 96 trials (24 per condition) in Experiments 1a and 1b and 48 (12 per condition) in Experiments 2 and 3 to reduce repetition of the same stimuli since participants had to combine both cues for their judgment.

### Stimuli

Male faces and voices were pre-selected based on their distance from the mean in trustworthiness (at least one standard deviation). In each experiment, 24 faces and 24 voices were used. For the faces in experiments 1a, 1b, and 2, we used artificial stimuli that parametrically increased or decreased the trustworthiness of a given face using a validated algorithm^11^. Each face identity was randomly matched with a voice identity and the value of its trustworthiness was chosen to match or oppose the voice. For example, if the voice was 1.25 standard deviations below the average trustworthiness and the condition was incongruent, then the image chosen was of that face identity manipulated to be 1.25 standard deviations above the mean. For Experiment 3, real faces from the Chicago Face Database were used^12^, which were pre-rated on trustworthiness. Then, white and Latino male faces one standard deviation above and below mean trustworthiness were selected and paired with voices as described above. For voices, there were no available databases, therefore we recorded our own. We recorded people reading a telephonic dialogue including the word “hi” (“Hola” in Spanish) 5 times. We collected 130 male voices (see Supplementary Information S1 for more details on the procedure followed to record them). A researcher blind to the hypotheses selected the best uttered “hi” for each. We then asked 53 people to rate the trustworthiness of each voice from 1 (not at all) to 9 (very trustworthy). Then, we selected voices that fell above and below the cutoff of one standard deviation to pair with the faces. Following evidence from past research^7^, voices judged as trustworthy had a significantly higher pitch than untrustworthy voices (*t*(22) = 3.86, *p* = 0.001, see Supplementary Table S1, Supplementary Figs. S1 and S2 for a summary of the acoustic properties of the voices).

### Procedure

**Figure 1:**
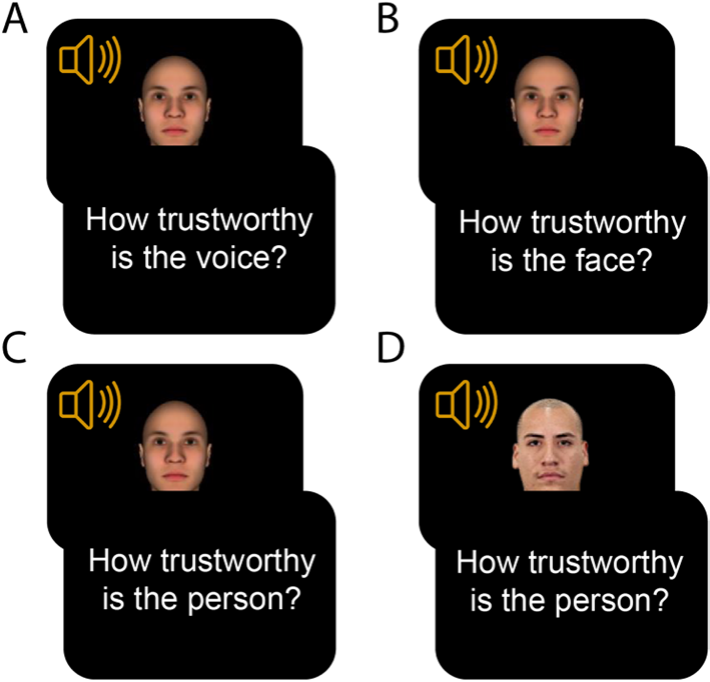
Experimental design. Participants were presented simultaneously with a face and a voice for 500 ms. Right after, participants reported, (**A**) the trustworthiness of the voice (Experiment 1A), (**B**) the trustworthiness of the face (Experiment 1B), or (**C**,**D**) the trustworthiness of the person for artificial (Experiment 2), and real faces (Experiment 3).

Participants were first told to use headphones during the experiment and were asked to test the sound system of their computer before starting the main task. After this, participants were instructed that they would have to rate the trustworthiness of voices (Experiment 1a), faces (Experiment 1b), or individuals (Experiments 2 and 3) on a scale from 1 to 9. Then, they completed the rating task. There was a 500ms fixation cross before every stimulus. Then, the face and the voice were simultaneously presented for 500 ms. The sound files varied in duration between 197 and 449 ms. They were matched in duration between conditions [*t*(22) = 0.502, *p* = 6.21, BF01 = 2.443]. Each sound file was presented only once and the face remained on the screen until the 500ms had passed. Immediately after, they were asked to report the trustworthiness of the voice (Experiment 1a), face (Experiment 1b), or individual (Experiments 2 & 3, see Fig. 1). There was no time limit to respond, and the next trial started immediately after with a fixation cross that lasted 500 ms. The task took on average 10–15 min to complete.

### Analyses

In all experiments, linear mixed-effect models were computed using the lme4^13^ and lmerTest^14^ packages in R. These predicted trustworthiness ratings based on the face and the voice’s trustworthiness, and the interaction between the two. The maximal effects structure that allowed for convergence across experiments included voices and faces as random slopes by participant, and voices and faces as random intercepts (except for Experiment 1a, where participants and voices were defined solely as random intercepts). Code and data for the analyses are available on OSF (see Data Availability statement). Trustworthiness levels were contrast coded as factors with -1 as low trustworthiness and 1 as high trustworthiness.

## Experiments 1a and 1b

We first tested whether participants’ assessments of trustworthiness in one modality were affected by the, in principle, ignored modality. We ran two experiments in which participants were asked to evaluate the trustworthiness solely of the voice (Experiment 1a) or the face (Experiment 1b). We hypothesized that, even though they were explicitly told to ignore one modality, participants would still encode and use it in their trustworthiness judgments.

### Participants

*Experiment 1a*: 40 participants were recruited. Two participants were removed because they did not finish the task, 11 failed the attentional check, and 1 provided the same answer on more than 80% of the trials. The final sample was 26 participants (11 women, average age 26.54 (8.03) years old).

*Experiment 1b:* 34 participants were recruited. Two participants were removed for incomplete responses and 7 failed the attentional check. No participants were removed for providing the same answer on more than 80% of the trials. The final sample was 25 participants (6 women, average age 27.48 (8.35) years old).

### Results

**Figure 2:**
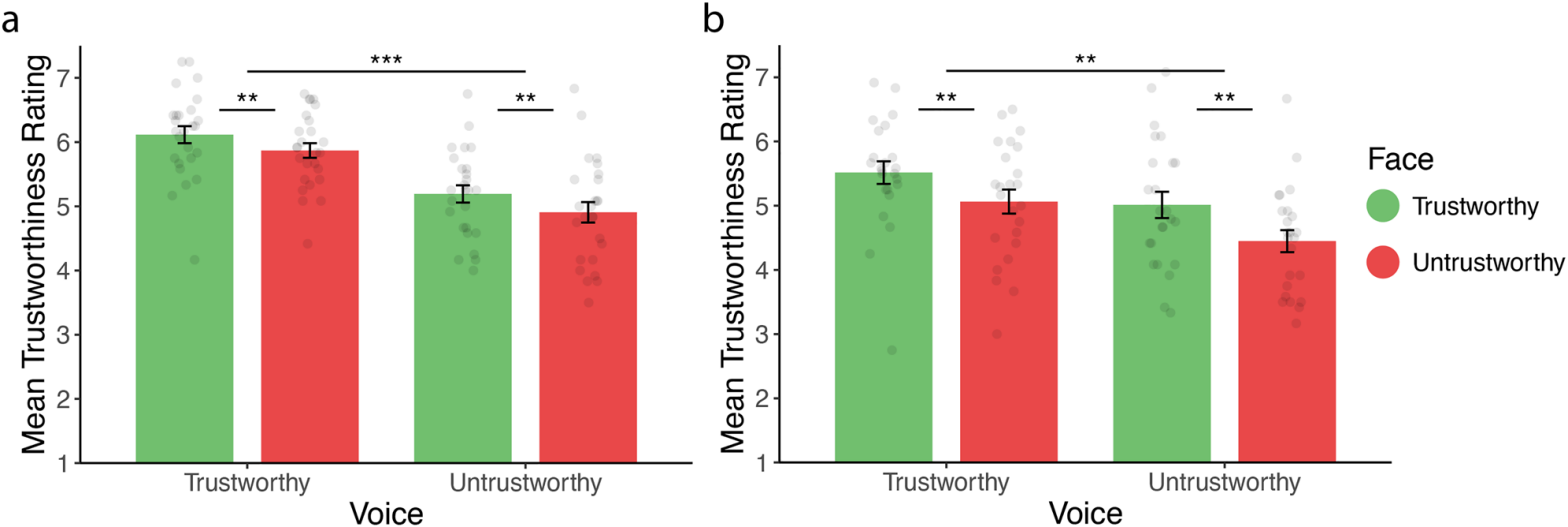
Mean trustworthiness ratings by face and voice category in Experiment 1a (**a**) and 1b (**b**). Error bars represent ± 1 standard error of the mean. **p < 0.01. *** p < 0.001.

*Experiment 1a:* On average, participants judged voices to be slightly trustworthy (mean rating = 5.52, SD = 0.52, t.test against mid-point (5): *t*(25) = 5.07, *p* < 0.001). Unsurprisingly, their judgments were affected by the trustworthiness of voices ($\beta =0.47 \left(0.09\right), p<0.001$, see Fig. 2a). Trustworthy voices were rated as more trustworthy than untrustworthy voices. As predicted, participants were also affected by the trustworthiness of faces, even though they were asked not to consider this cue in their judgment ($\beta =0.13 \left(0.04\right), p=0.001$). Voices drove judgments irrespective of faces' trustworthiness, and vice versa (interaction: $\beta =- 0.01 \left(0.04\right), p=0.81$). Overall, participants could not avoid being affected by faces when they were judging the trustworthiness of voices.

*Experiment 1b*: On average, participants judged faces as neutral (mean rating = 5.01, SD = 0.74, t. test against mid-point (5): *t*(23) = 53.25, *p* = 0.95). Unsurprisingly, their judgments were affected by the trustworthiness of faces ($\beta =0.25 \left(0.07\right), p= 0.001,$ see Fig. 2b). Participants were also affected by voice trustworthiness, even though they were asked to judge only faces ($\beta =0.34 \left(0.10\right), p= 0.001).$ Again, we failed to find an interaction: faces drove judgments irrespective of voices' trustworthiness, and vice versa (interaction: $\beta =-0.02 \left(0.04\right), p=0.59$). Overall, participants could not prevent voices from weighing in on their judgment of faces.

## Experiment 2

Faces and voices are weighted in trustworthiness first impressions even when people are asked to filter them. Across Experiments 1a and 1b, voices seemed to drive impressions more than faces. In Experiments 2 and 3, we explore how both modalities are combined when judging the individual.

### Participants

Forty-nine participants were recruited. Two participants were removed for incomplete answers, five for failing the attentional check, and one for providing the same answer on more than 80% of the trials. The final sample was 39 participants (13 women, average age 27.49 (8.83) years old).

### Results

**Figure 3:**
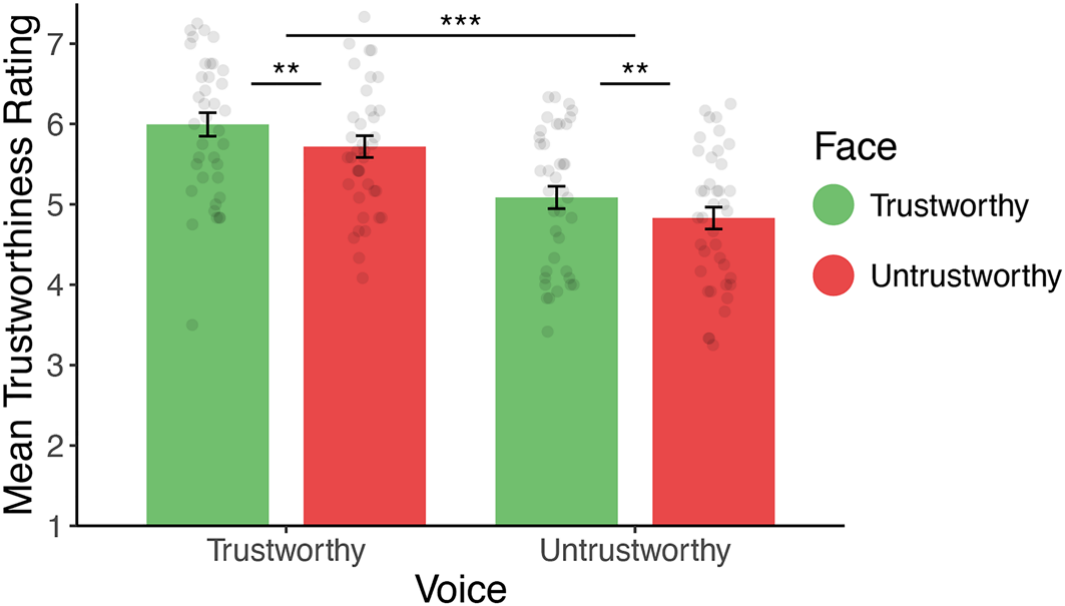
Mean trustworthiness ratings by face and voice category in Experiment 2. Error bars represent ± 1 standard error of the mean. **p < 0.01. ***p < 0.001.

On average, participants rated individuals as slightly trustworthy (mean rating = 5.41, SD = 0.73, t. test against mid-point (5): *t*(38) = 3.50, *p* = 0.001). Trustworthiness judgments were predicted by the trustworthiness of the face and the voice (face: $\beta =0.13 \left(0.04\right), p= 0.001$; voice: $\beta =0.45 \left(0.09\right), p< 0.001,$ see Fig. 3), with no interaction ($\beta =0.0 \left(0.04\right), p= 0.90$). Conceptually replicating results from Experiments 1a and 1b, trustworthiness judgments were more sensitive to the voice than the face of the individual. Across Experiments 1 and 2, judgments of voices overpowered those of faces.

## Experiment 3

Across two experiments, we find that people are irremediably affected by the presence of voices and faces even when asked to ignore one or the other. Furthermore, voices appear to impose a higher bias than faces in trustworthiness judgments, even when they were matched on levels of trustworthiness. There was an important difference between stimuli, however: the auditory stimuli were real, while the visual ones were artificial. This could explain the bias people showed towards voices, especially considering that people overweigh what is more perceptually salient, that is, more distinct and unique. Hence, in Experiment 3, we used a set of real faces to test whether the voice bias remains.

### Participants

Fifty-eight participants were recruited. Six participants were removed for incomplete data, nine failed the attentional check, and one provided the same answer on more than 80% of the trials. The final sample was 42 participants (14 women, average age 24.61 (6.88) years old).

### Results

**Figure 4:**
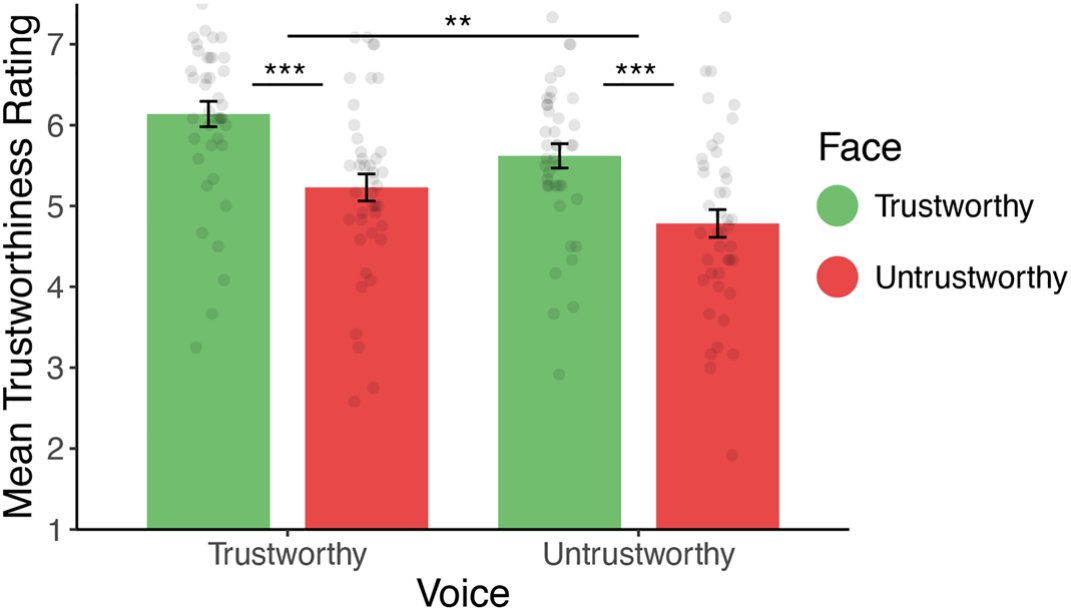
Mean trustworthiness ratings by face and voice category in Experiment 3. Error bars represent ± 1 standard error of the mean.﻿ **p < 0.01. *** < 0.001.

On average, participants rated individuals as trustworthy (mean rating = 5.44, SD = 0.93, t.test against mid-point (5): *t*(41) = 3.08, *p* = 0.004). Again, trustworthiness ratings were significantly predicted by the trustworthiness of the face and the voice (face: $\beta =0.44 \left(0.08\right), p< 0.001$; voice: $\beta =0.24 \left(0.05\right), p< 0.001$, see Fig. 4), with no interaction between them ($\beta =0.02 \left(0.03\right), p= 0.58$). This time, however, trustworthiness ratings were better predicted by face than voice trustworthiness, showing that changing the stimulus set affected the weight participants gave to visual cues (see Table 1 for a summary of results).

**Table 1:** Summary of results.

Experiment 1a		Experiment 1b	
Coefficient $(\upbeta )$	Estimate (SE)	Coefficient $(\upbeta )$	Estimate (SE)
Trustworthiness of voices (Experiment 1a) and faces (Experiment 1b)			
Voices	0.47 (0.09)***	Voices	0.34 (0.10)**
Faces	0.13 (0.04)**	Faces	0.25 (0.07)**
Voices × faces	− 0.01 (0.04)	Voices × faces	− 0.02 (0.04)

Experiment 2		Experiment 3	
Coefficient $(\upbeta )$	Estimate (SE)	Coefficient $(\upbeta )$	Estimate (SE)
Trustworthiness of a person with artificial (Experiment 2) and real (Experiment 3) faces			
Voices	0.45 (0.09)***	Voices	0.24 (0.05)**
Faces	0.13 (0.04)**	Faces	0.44 (0.08)***
Voices × faces	0.00 (0.04)	Voices × faces	0.02 (0.03)

Regression coefficients of all experiments where voices and faces are contrasted coded (untrustworthiness = − 1, trustworthiness = 1). **p < 0.01. ***p < 0.001.

To further explore why for this set of stimuli faces were more important than voices in driving participants’ judgments, we asked a new set of participants (N = 9) to rate the similarity of pairs of faces and voices used in Experiment 3 on a scale from 1 (not similar) to 9 (very similar). We hypothesized that, in contrast to Experiments 1 and 2 where faces were very similar, faces in Experiment 3 would be judged as more dissimilar than voices, which could explain why they had a stronger impact on judgments. To test this, we first computed a similarity index by averaging the rating that each face and voice received when compared with the other faces and voices. As expected, faces were significantly judged as less similar than voices (mean similarity faces = 3.13, SD = 0.34, mean similarity voices = 4.53, SD = 0.38, *t*(41) = 3.08, *p* < 0.001). To further explore the impact of stimulus distinctiveness on trustworthiness judgments, we computed the difference between the average similarity of the voice and the average similarity of the face for each trial. Positive values mean that the face is more distinct and negative values mean that the voice is more distinct. The original effect of voice was modulated by this distinctiveness index, as evidenced by a significant interaction ($\beta =-0.15 \left(0.07\right), p= 0.04)$, indicating that voices had a reduced impact when paired with distinct faces, while their impact increased when voices were more distinct than faces. Stimuli distinctiveness did not modulate the impact of faces ($\beta =0.04 \left(0.09\right), p= 0.66)$, probably as a consequence of their larger distinctiveness in the stimulus set.

## Discussion

In human interactions, we receive complex multidimensional information about the other person. Combining several cues to form first impressions is thus fundamental. Although increasingly studied (e.g.,^10^), exactly how we integrate these cues is still an open question, especially when these come from different sensory modalities. Across four experiments, we found that people combine visual (face) and auditory (voice) cues in an automatic and rapid way to form trustworthiness impressions. Furthermore, people were incapable of filtering the other sensory modality when asked to judge based only on the face or the voice (Experiments 1a and 1b). When considering which modality was more important in driving trustworthiness judgments, we found that it depends on the set of stimuli used. For the first three experiments, conducted with artificial faces (perceptually very similar to each other) and real voices (more different), the voice was the main determiner of trustworthiness judgments. However, when artificial faces were replaced with real faces, which were perceptually more dissimilar, results changed: trustworthiness judgments were better predicted by faces than voices. Consistently throughout all four experiments, there was no interaction between face and voice valence affecting trustworthiness judgments.

Attention vastly affects the multisensory integration of voices and faces to inform trustworthiness. First, when asked to focus only on one sensory modality, trustworthiness judgments were mostly driven by the target dimension. However, participants were not able to fully block out information from the unattended channel. This inability suggests that multisensory integration during trustworthiness judgments is relatively automatic. Second, when questioning which modality trumps the other and is thus given priority when judging trustworthiness, we found interesting results. In prior studies, the relative contribution of each modality depended on the trait under investigation. For example, while faces matter more when judging trustworthiness^9,10^, voices become more important when judging dominance^1^. In our case, voices took precedence when combined with artificial faces, while the reverse was true when using real faces. We understand this divergence in terms of a relatively low-level feature: the distinctiveness of items within their set. This suggests that people prioritize—whether implicitly or explicitly—one modality or another in a flexible manner. This weighing of information depends on the features of the context, such as saliency driven by stimulus distinctiveness. Our results also suggest that first impressions—unsurprisingly—follow fundamental principles of perception and attention, and one needs to be cautious when drawing conclusions about sensory prioritization without controlling for low-level features that drive attention. Future studies could provide a better understanding of the role of both attention and low-level features in personality judgments. For example, looking into different time-tables to assess the role of automaticity and timing and whether noisy signals (e.g., auditory noise or reduced visual contrast) help modulate the relative saliency of each modality.

An alternative explanation for the difference in the weighting of voices and faces between experiments 3 and 4 relates to the naturalness of the stimuli. It is possible that people have more difficulty judging artificial faces, an effect that might be exacerbated by the short presentation time. Additionally, people may have somewhat discredited the artificial faces as they are not attached to real people and are therefore by definition less informative. In other words, artificial faces are useful for judgments in which no other information is available, but if you have a more dependable source—namely, a real voice from a real person who has some intrinsic trustworthiness—then this cue loses some of its value.

Faces and voices are combined to inform trustworthiness judgments^1,9,10^. Across four experiments, we have failed to find evidence for an interaction between modalities, suggesting that their combination is additive. We should avoid hasty conclusions about a lack of crosstalk between modalities in trustworthiness judgments. The lack of interaction here might be the consequence of our experimental design. Importantly, we only used stimuli at least one standard deviation away from mean trustworthiness, and each face was presented with a voice with an equivalent level of (un)trustworthiness. This restricted range might have mathematically masked the crosstalk between modalities and imposed a perfect additive model, with stimuli perfectly adding to or canceling each other out. Caution also needs to be had regarding the unnatural stimuli used in the experiments: the faces remained static while their voices were playing. Further research needs to be conducted to address this issue using more naturalistic stimuli.

An important limitation of our study is that all the stimuli were from white or Latino males. We decided to restrict the stimuli this way to control for other important features related to gender and race as well as ingroup/outgroup belonging—particularly since participants were all Spanish. For instance, it has been shown that ethnic cues conveyed by both facial (i.e., ethnic phenotypes) and vocal (i.e., accent) features jointly influence intergroup impressions, with people being more affected by others’ voices^15,16^. Face-voice combination also influences interpersonal impressions^1,9,17^. Future research could explore the possible interaction—or lack thereof—between modalities and expand the range of stimuli to make the results more generalizable to other genders and backgrounds. On the one hand, we need to explore the degree to which the lack of interaction is caused by our experimental design, and, on the other, whether the crosstalk appears once a wider range of more diverse stimuli is used.

## Conclusion

Social cognition requires combining a vast array of multimodal information. The study of person perception has been traditionally fragmented, with little effort in putting the pieces together. Here, we have explored the impact of voices and faces on trustworthiness judgments to demonstrate the simplicity and automaticity with which such a combination of sensory sources occurs. This is a first step towards the goal of unifying these fragments, in an attempt to experimentally capture the complexity of our social environment. As a next step, if we understand how this applies to minorities, we might be able to find ways to tilt the scale in ways that naturally distrusted people can be more easily trusted.

### Supplementary Information

Supplementary Information.

## Data Availability

All data and code used for analyses are available at https://osf.io/tjqvw.
