# Supplementary material for: Automating weighing of faces and voices based on cue saliency in trustworthiness impressions
Source: Sci Rep. 2023 Nov 16;13:20037. doi: 10.1038/s41598-023-45471-y (PMC10654569; doi:10.1038/s41598-023-45471-y)

**SUPPLEMENTARY INFORMATION**

**Automating weighing of faces and voices based on cue saliency in trustworthiness impressions**

**SUPPLEMENTARY METHODS**

*Recording method*. Voices were recorded using the following script (in order to elicit more naturalistic/contextualized instances of the word “Hola”):

A: ¡Hola! Soy Pablo. ¿Quién habla?

B: ¡Hola, Pablo! Habla Pedro.

A: Ah, hola. ¿Está Juan?

B: Sí, ahora te paso.

C: Hola Pablo.

A: Hola Juan, ¿cómo estás?”

**SUPPLEMENTARY TABLES & FIGURES**

**Supplementary Table 1 | Properties of voices split by trustworthiness levels.**

| Voice | F0 | Intonation | Dispersion | HNR | Duration | Rating | Reliability |
| --- | --- | --- | --- | --- | --- | --- | --- |
| Trustworthy | 156.92 (28.28) | 69.67 (83.83) | 746.97 (61.34) | 6.15  (2.52) | 0.29  (0.05) | 6.32  (0.08) | 0.64 |
| Untrustworthy | 118.68 (19.42) | 28.70 (13.70) | 732.93 (48.01) | 6.45  (2.26) | 0.31  (0.07) | 4.77  (0.12) | 0.60 |
|  | 38.24** | 40.97 | 14.04 | -0.3 | -0.02 | 1.55*** | 0.04 |

*Note. Average of mean fundamental frequency (in Hz), intonation (in dB), formant dispersion (a ratio between consecutive formant means), harmonic-to-noise ratio (a ratio between the strength of the harmonic component relative to the nonharmonic component), duration (in seconds), trustworthiness ratings (from 1 to 9) and reliability (Cronbach’s alpha) of the 24 voices used (12 for trustworthiness level). Standard deviation is in parenthesis, and differences for each property between voice types are shown in the last row. **p<0.01. ***p<0.001.*

**Supplementary Figure 1 | Acoustic properties of voices broken by high/low trustworthiness.**


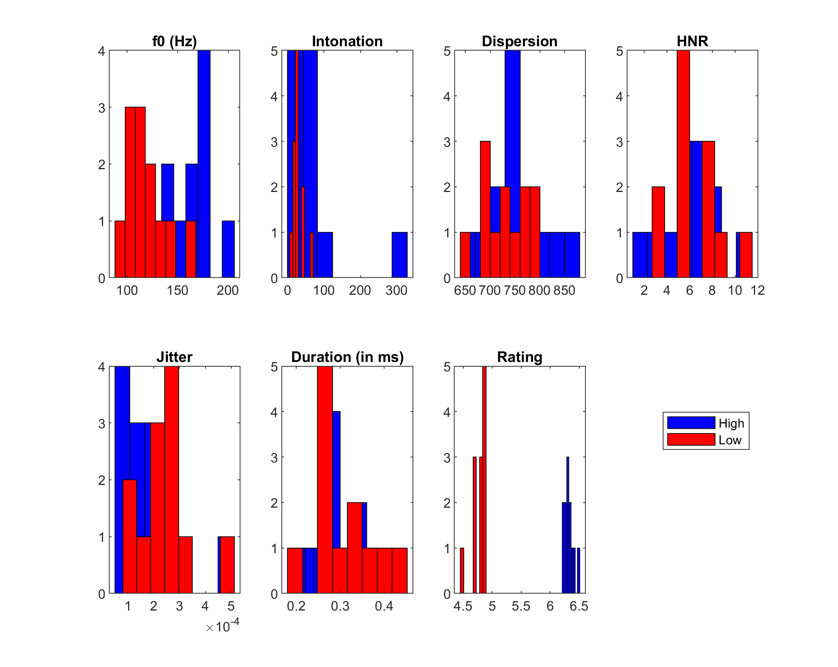


**Supplementary Figure 2 | Spectrogram of voices divided by trustworthiness level.** Voices rated as high trustworthiness (left panel) had a higher pitch than voices rated as low trustworthiness (right panel):


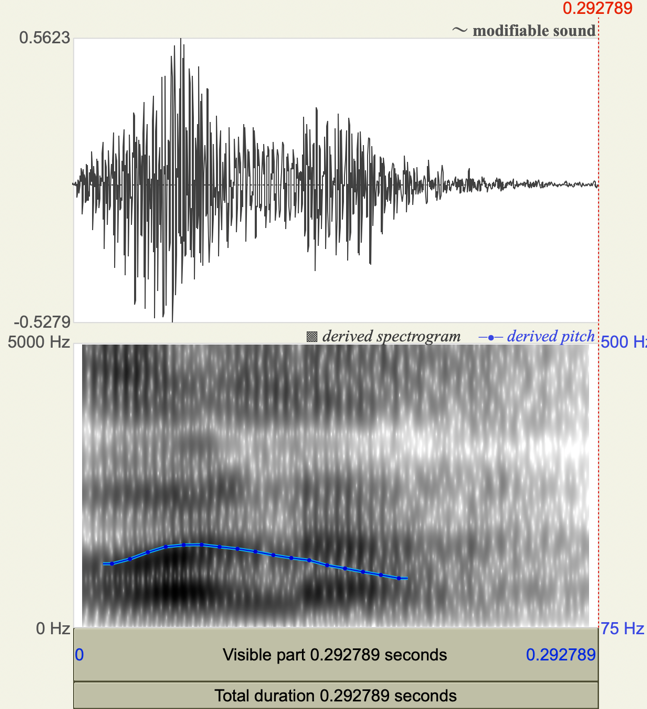

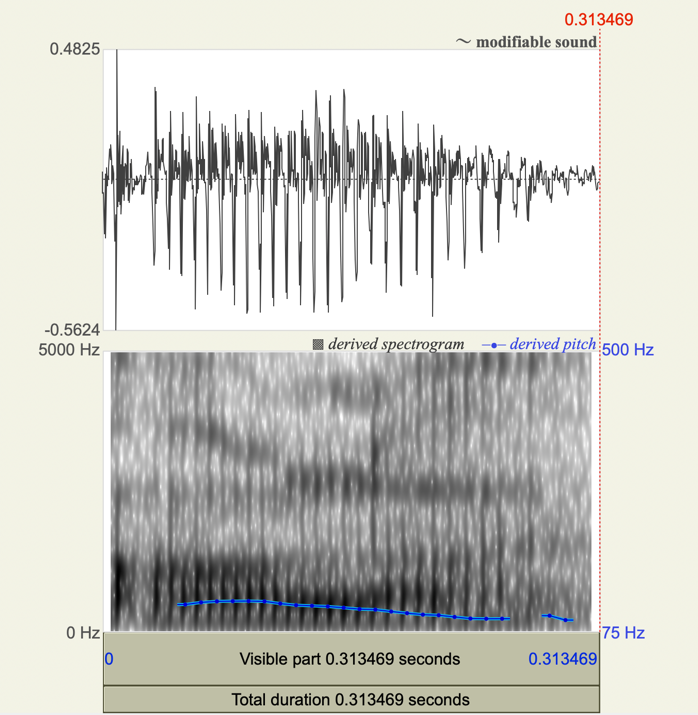

Supplement: Supplementary file 1 — Supplementary Information. [file 41598_2023_45471_MOESM1_ESM.docx]
